# RNA-Seq Reveals miRNA and mRNA Co-regulate Muscle Differentiation in Fetal Leizhou Goats

**DOI:** 10.3389/fvets.2022.829769

**Published:** 2022-03-25

**Authors:** Junning Ye, Xiuhui Zhao, Huiwen Xue, Xian Zou, Guangbin Liu, Ming Deng, Baoli Sun, Yongqing Guo, Dewu Liu, Yaokun Li

**Affiliations:** ^1^College of Animal Science, South China Agricultural University, Guangzhou, China; ^2^State Key Laboratory of Livestock and Poultry Breeding, Guangdong Key Laboratory of Animal Breeding and Nutrition, Institute of Animal Science, Guangdong Academy of Agricultural Sciences, Guangzhou, China; ^3^National Local Joint Engineering Research Center of Livestock and Poultry, South China Agricultural University, Guangzhou, China

**Keywords:** Leizhou goat, muscle differentiation, miRNA, mRNA, enrichment

## Abstract

Muscle differentiation is an essential link in animal growth and development, and microRNA and mRNA are indispensable in skeletal muscle differentiation. To improve the meat quality and production of the Leizhou goat, it is vital to understand the molecular mechanism by which its skeletal muscle differentiates. By RNA sequencing (RNA-SEQ), we established miRNA-mRNA profiles of Leizhou goats at three stages: fetal day 70, 90, and 120. There were 991 differently expressed mRNAs and 39 differentially expressed miRNAs found, with the differentially expressed mRNAs mainly enriched in calcium ion binding, ECM-receptor interaction, and Focal adhesion. CKM and MYH3, two muscle differentiation markers, were significantly differentially expressed during this period. In addition, we found that chi-miR-129-5p, chi-miR-433, and chi-miR-24-3p co-regulate muscle differentiation with their target genes. Finally, we can confirm that muscle differentiation occurred in Leizhou goat between 90 and 120 days of the fetus. This study is helpful to better explore the molecular mechanism of goat muscle differentiation.

## Introduction

In recent years, China's mutton industry has developed rapidly, especially since the outbreak of the African Swine Fever in 2018. Goat is an essential agricultural animal, and its meat has a unique flavor. Compared with other domestic animals, goat meat has more protein and lower fat and cholesterol contents (1). Goats are mainly used for meat production in most areas. Studies have shown that skeletal muscle accounts for 40% of body weight (2). Therefore, understanding the molecular differentiation and development mechanism of skeletal muscle in goats is very important for biology and the agricultural economy.

Myogenesis is a complicated and closely controlled process involving embryonic precursors to the myogenic lineage, myoblast growth, and cell cycle exit. Mononucleated precursor cells fuse to generate multinucleated mature myotubes in the final stage (3, 4). Myogenic regulatory factors (MRFs), such as Myf5, MyoD, Myf6, and myogenin, which play a critical role in muscle development, have been found to govern this sequence of activities in previous research. Myf5 and MyoD are involved in controlling myoblast cell production, proliferation, and lifespan, whereas Myf6 and myogenin are involved in the last phases of differentiation (5, 6).

MicroRNAs (miRNAs) are a class of short non-coding RNAs (22 nt) that bind to partly complementary regions on target mRNAs to control gene expression post-transcriptionally (7), which play vital roles in many biological processes that regulate development, cell differentiation, and disease processes (8). MiRNAs bind miRNA “seed sequences” (nucleotides 2–7) to the 3'untranslated region (UTR) of the target mRNA through complementary base pairs (9), degrading or destabilizing RNA information or inhibiting protein translation, depending on the number of complementary base pair matches or the number of miRNA target sites within the 3'UTR (10). MiRNAs are also essential for satellite cell function and muscle development, according to muscle-specific ablation of Dicer, a critical enzyme for the generation of precursor (pre)miRNAs (11). RNA-SEQ has been widely employed in recent years to investigate the genetic mechanisms of skeletal muscle growth and development in various domestic animals (12, 13). Muscle-specific miRNAs, such as miR-1, miR-133, and miR-206, have been found to play an essential role in the proliferation, differentiation, and regeneration of skeletal muscle cells in studies (14–16). MiR-101a was discovered to be concentrated in goat skeletal muscle and up-regulated during goat SMSC (Skeletal Muscle Satellite Cells) differentiation, according to Li's result. More myotubules were formed, and myoblast gene expression was greater when miR-101a was overexpressed (17). MiR-487b-3p is important for myogenic proliferation and differentiation, according to Wang's findings, and can suppress C2C12 myoblast proliferation, differentiation, and myogenesis (18). More miRNAs have been implicated in muscle differentiation as research has progressed in recent years. MiR-320-3p was discovered to promote F-actin accumulation by inhibiting CFL2, activating YAP1, and increasing myoblast proliferation, ultimately compromising myoblast differentiation (19). MiR-22-3p suppresses skeletal muscle cell proliferation while promoting cell differentiation in Hu sheep by targeting IGFBP3 (20). MiR-499-3p can be sponged by circPTPN4 to modulate NAMPT expression, boosting myoblast proliferation and triggering myogenic differentiation (21). MiR-100 inhibits IGF1R/PI3K/AKT signaling in bovine skeletal muscle cells, resulting in reduced differentiation and protein expression (22). MiR-29c was sponged by circUSP13, which increased IGF1 expression and up-regulated MYOG and MYHC expression, promoting differentiation of goat primary myoblasts and inhibiting apoptosis (23).

Furthermore, miRNAs can influence muscle fiber change and metabolism. Tnni1 and Tnni2 are two genes that are usually thought to be marker genes for slow and rapid muscle fibers, respectively. Gan et al. discovered that overexpression of miR-499 in mouse skeletal muscle tissue resulted in considerable upregulation of Tnni1 gene expression and downregulation of Tnni2 gene expression, indicating that miR-499 is involved in skeletal muscle fast and slow muscle fiber transition (24). MiR-199-3p targeting TEAD1 was able to increase the HMB-induced changeover between slow and fast muscle fiber types in response to the production of beta-hydroxy-beta-methylbutyrate (HMB) (25). MiR-130b, by directly targeting the long non-coding RNA MyHC IIA 3'-UTR, can regulate myogenesis and preserve the rapid muscle fiber phenotype (26). MiRNAs are also thought to have a role in aberrant muscle growth. Eisenberg discovered 185 variations in miRNA expression in 10 different types of muscular dysfunction (27). Mccarthy et al. were the first to show that miRNAs play an important role in skeletal muscle growth via functional overload induction (28).

There is a high demand for goat meat in China, and a huge amount of goat meat must be imported each year. The Leizhou goat is found mostly in Guangdong Province, China, in the Leizhou Peninsula. It is an exceptional native goat breed in Guangdong Province, with qualities such as harsh feeding, resilience to high temperatures and humidity, strong disease resistance, early sexual maturity, and superior meat quality. However, due to a long-term absence of efficient conservation measures, inbreeding, and inadequate breeding management procedures, the breed has degraded substantially and become significantly smaller. Secondary myogenesis is the process through which fetal progenitor cells fuse to generate secondary fibers, which is essential for fetal skeletal muscle development. Secondary myogenesis is essential for muscle growth and maturation (fibrous type) during embryonic development, but there is no net increase in the number of muscle fibers after birth (29, 30). As a result, knowing the molecular process of skeletal muscle synthesis in pregnant Leizhou goats is crucial (31). Using the RNA-SEQ method, we investigated the transcriptome profiles of Leizhou goat fetuses' Longissimus dorsi at 70, 90, and 120 days, incorporating previous work on goat skeletal muscle. The differentially expressed genes (DEGs) in the longissimus dorsi of Leizhou goat fetuses were found during three periods. DEGs were tested for their ability to enrich gene ontology (GO) and Kyoto Encyclopedia of Genes and Genomes (KEGG) pathways. This is the first transcriptome sequencing of a Leizhou goat's longest dorsal muscle to reveal the molecular mechanism of muscle differentiation and provide options for increasing goat meat production and quality.

## Materials and Methods

### Sample Collection

Nine healthy pregnant Leizhou goats (Carpa hircus) were obtained from a goat farm in Leizhou, Guangdong province. The animals were divided into three groups and slaughtered at 70, 90, and 120 days of gestation. The longissimus dorsi of the fetus was quickly taken out, frozen in liquid nitrogen, and returned to the laboratory for RNA extraction.

### cDNA Library Preparation

Total RNA was extracted and processed with DNase I using a TRIzol reagent (Invitrogen, Carlsbad, CA, USA) (Qiagen, Beijing, China). For RNA degradation and contamination, we employed a 1% agarose gel electrophoresis and the Nanophotometer® Spectrophotometer (IMPLEN, CA, USA) to determine RNA purity. In a Qubit® 2.0 Fluorometer, the RNA concentration was determined using the Qubit® RNA Assay Kit (Life Technologies, CA, USA). The Agilent Bioanalyzer 2100 system was used to test RNA integrity using the RNA Nano 6000 Assay Kit (Agilent Technologies, CA, USA). The tiny RNA library received 3 μg total RNA per sample as input material. NEBNext® Multiplex Small RNA Library Prep Set for Illumina® (NEB, USA.) was used to produce sequencing libraries, and index codes were applied to assign sequences to each sample, as per the manufacturer's guidelines. NEB 3′ SR Adaptor was ligated to the 3′ end of miRNA, siRNA, and piRNA directly and explicitly. The excess of 3′ SR Adaptor (that remained free after the 3′ ligation reaction) hybridized to the SR RT Primer after the 3′ ligation reaction. It made a double-stranded DNA molecule out of the single-stranded DNA adaptor. Furthermore, dsDNA does not ligate to the 5′ SR Adaptor in the following ligation phase because it is not a T4 RNA Ligase 1-mediated ligation substrate. The miRNA, siRNA, and piRNA 5'ends adapter was ligated to their 5'ends. After that, M-MuLV Reverse Transcriptase (RNAse H-) was used to make first-strand cDNA. LongAmp Taq 2X Master Mix, SR Primer for Illumina, and index (X) primers were used to achieve PCR amplification. PCR products were purified on an 8 percent polyacrylamide gel (100V, 80 min). DNA fragments of 140 to 160 bp (the length of short non-coding RNA plus the 3′ and 5′ adaptors) were recovered and dissolved in an 8-liter elution solution. Finally, DNA High Sensitivity Chips were used to test library quality on the Agilent Bioanalyzer 2100 system.

### Sequencing and Transcriptome Assembly

The index-coded samples were clustered using the TruSeq SR Cluster Kit v3-cBot-HS on a cBot Cluster Generation System, according to the manufacturer's instructions (Illumina). After cluster creation, the library preparations were sequenced on an Illumina Hiseq 2500/2000 platform, and 50 bp single-end reads were produced. Raw fastq data (raw readings) were initially processed using proprietary Perl and Python programs. Clean data (clean reads) were acquired in this stage by deleting ploy-N reads, reads with 5′ adapter contamination, reads without the 3′ adapter or the insert tag, reads with ploy A, T, G, or C, and poor quality reads from raw data. We have the following stages in data processing to produce high-quality readings: delete reads with a quality value of sQ = 20 that account for more than 30% of the whole read, and remove reads with a ratio of N more than 10%. The tiny RNA tags were then mapped to the Carpa Hircus reference sequence without mismatch using Bowtie (version: Botie-0.12.9, main parameter: -v 0 -k 1) (32) to examine their expression and distribution on the reference.

### Prediction of the Differentially Expressed MRNAs and MiRNAs

To find known miRNA, mapped small RNA tags were employed before prediction. To get the possible miRNA and draw the secondary structures, we utilized miRBase20.0 as a reference, customized software mirdeep2 (33), and sRNA-tools-cli. MiRNA counts and base bias were calculated using custom scripts on the first location of the identified miRNA with a certain length and on all identified miRNA positions. Small RNA tags were mapped to RepeatMasker, Rfam database, or data from the selected species to eliminate tags coming from protein-coding genes, repeat sequences, rRNA, tRNA, snRNA, and snoRNA. The properties of miRNA precursors' hairpin structure can be exploited to predict new miRNA. The existing software miREvo (34) and mirdeep2 (33) were used to predict new miRNA by examining the secondary structure, Dicer cleavage site, and minimal free energy of the short RNA tags that had not been annotated in the previous phases. Custom scripts were utilized simultaneously to acquire the detected miRNA counts and base bias on the first location with a certain length and on each position of all identified miRNA, respectively.

### Target Gene Prediction

MiRanda (version: miRanda-3.3a, main parameter: -sc 140 –en 10 –scale 4 –strict –out) (35) was used to predict the target gene of miRNA in animals.

### GO and KEGG Enrichment Analysis

On the target gene candidates of differentially expressed miRNAs, we used Gene Ontology (GO) enrichment analysis (“target gene candidates” in the following). For the GO enrichment analysis, a GOseq-based Wallenius non-central hypergeometric distribution (36) was used, which may account for gene length bias. KEGG (37) is a database resource for deducing high-level functions and utilities of biological systems like the cell, organism, and ecosystem from molecular-level data, particularly large-scale molecular datasets generated by genome sequencing and other high-throughput experimental technologies (http://www.genome.jp/kegg/). The statistical enrichment of target gene candidates in KEGG pathways was tested using the KOBAS (38) program.

### Quantitative Real-Time Polymerase Chain Reaction Validation

For the qRT-PCR study, 1 μg of total RNA was reverse transcribed according to the manufacturer's instructions using the RT reagent Kits with gDNA Eraser (Takara, Dalian, China). The qRT-PCR was carried out using a StepOnePlus Real-Time PCR System (Life Technologies, Gaithersburg, MD, USA) using Fast Start Universal SYBR Green Master (ROX) according to routine procedures (Roche, Mannheim, Germany).

## Results

### Read Mapping

In total, 107,092,006, 87,993,093, 79,546,141, 85,410,197, 80,991,312, 81,045,992, 84,349,188, 74,773,152, and 89,449,091 mapped reads were obtained from the clean data from M70-1, M70-2, M70-3, M90-1, M90-2, M90-3, M120-1, M120-2, and M120-3 libraries, and more than 93% were mapped to the Capra hircus reference genome (Table 1). Additionally, in our study, the proportion of nucleotides with Q30 of each sample was >92%.

**Table 1:** Summary of clean reads mapped to the Capra hircus reference genome.

**Sample**	**M70-1**	**M70-2**	**M70-3**	**M90-1**	**M90-2**	**M90-3**
Raw reads	112748682	93168264	86268494	92489296	88219642	8821485
Clean reads	111282754	91680592	83389986	89435456	84788828	85471184
Q30 (%)	92.91	93.94	93.41	93.12	92.87	93.08
GC content (%)	54.4	57.34	52.25	54.35	52.15	55.63
Total mapped	107092006 (96.23%)	87993093 (95.98%)	79546141 (95.39%)	85410197 (95.5%)	80991312 (95.52%)	81045992 (94.82%)
**Sample**	**M120-1**		**M120-2**		**M120-3**	
Raw reads	93831684		82736310		97156680	
Clean reads	89851232		79494532		94138450	
Q30 (%)	93.77		92.99		93.13	
GC content (%)	60.03		55.92		55.45	
Total mapped	84349188 (93.88%)		74773152 (94.06%)		89449091 (95.02%)	

*M70, M90, and M120 goat fetuses*.

### Enrichment Analysis of the Differentially Expressed MRNAs

**Figure 1:**
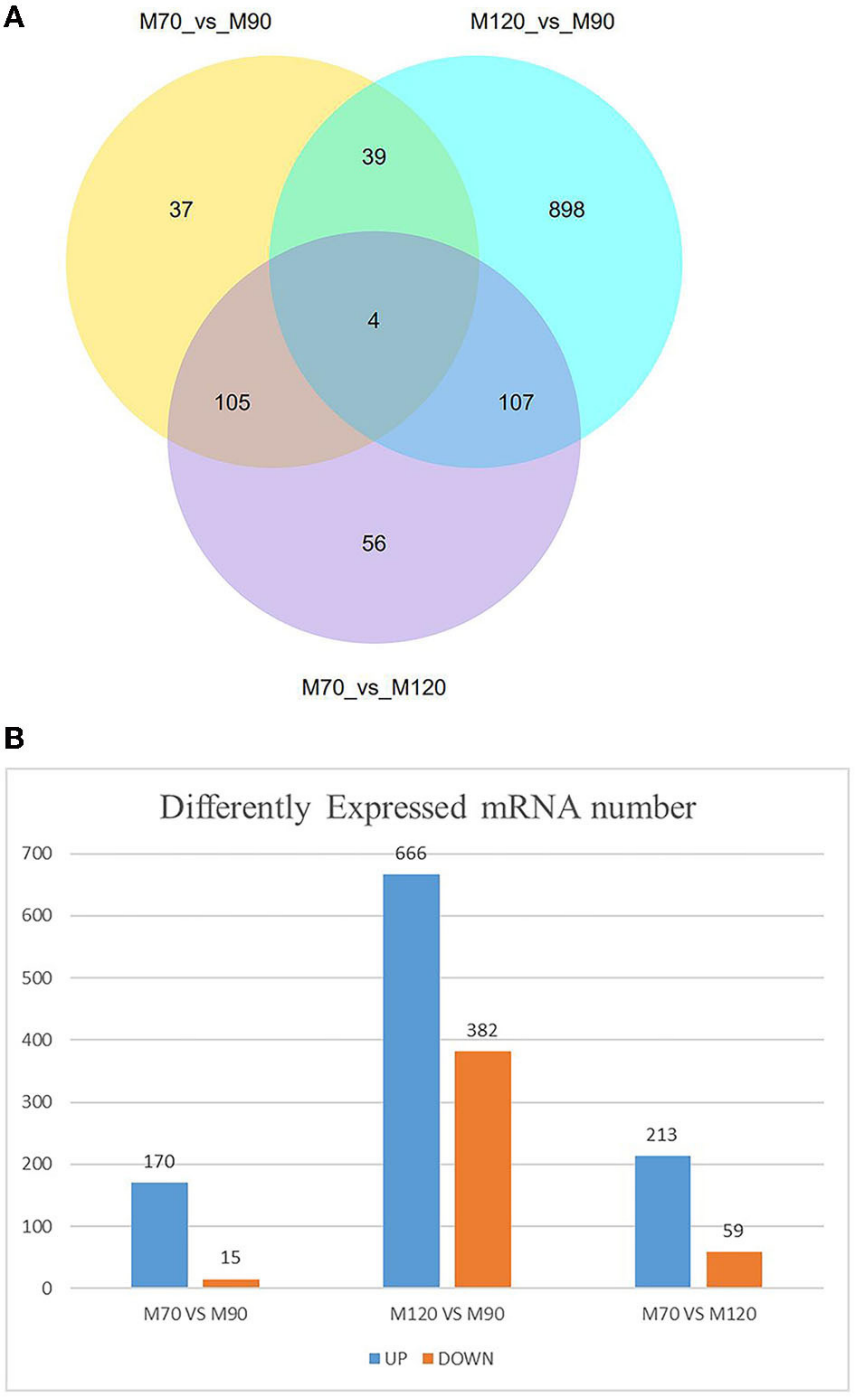
Number of DEGs at different time stage comparisons. **(A)** The venn diagram shows the number of DEGs across three comparisons: M70 VS M90, M120 VS M90, M70 VS M120. **(B)** The number of up- and downregulated DEGs across three comparisons: M70 VS M90, M120 VS M90, M70 VS M120, with the threshold set to q < 0.05.

**Figure 2:**
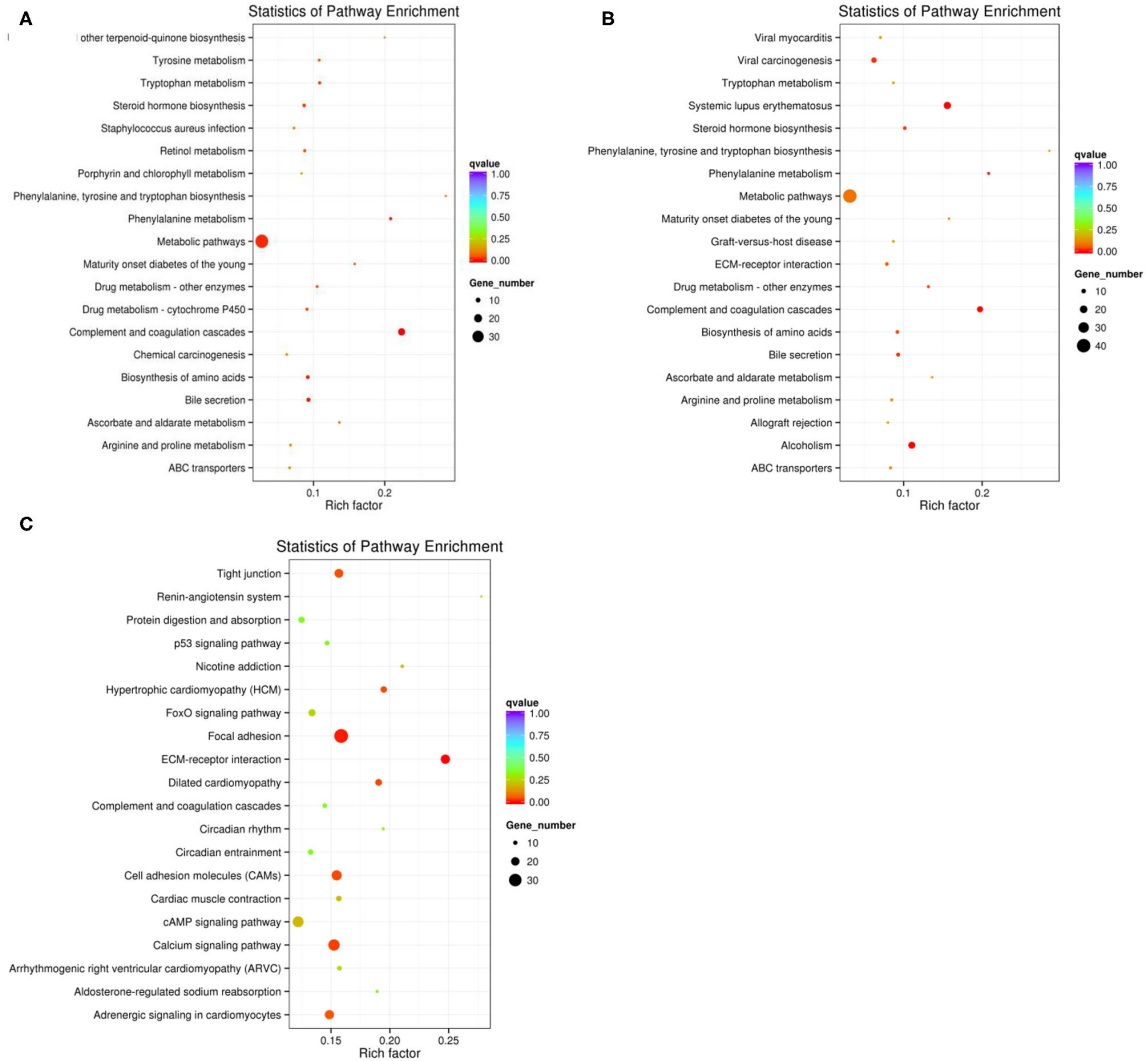
**(A)** The top KEGG enrichment analyses of the differentially expressed mRNAs in M70 vs. M90. **(B)** in M70 vs. M120. **(C)** in M120 vs. M90.

When p < 0.05, the transcripts can be classified as differential expressions, we discovered that 991 mRNA were differentially expressed. Compared with the M70 group, there were 170 up-regulation and 15 down-regulation in the M90 group, and 213 up-regulation and 59 down-regulation in the M120 group, respectively. Compared with the M90 group, 666 and 382 mRNAs were, respectively up-regulated and downregulated in the M120 group (Figures 1A,B). GO and KEGG enrichment analyses were performed further to demonstrate the biological functions of differentially expressed mRNAs. We found 106 GO terms with P < 0.05. Most of the M70 vs. M90 group were related to cellular amino acid decomposition processes, various peptidase activities, and peptidase inhibition activities (Supplementary Material 1). The M70 vs. M120 group was mainly enriched in blood coagulation, protein dimerization activity, and extracellular region (Supplementary Material 2). The M120 vs. M90 group is mainly enriched in cell response, cell adhesion, and calcium ion binding (Table 2 and Supplementary Material 3). The M70 vs. M90 and M70 vs. M120 groups had similar enrichment results in KEGG pathways, mainly in the metabolic pathways, complement, and coagulation cascades (Figure 2A). M70 vs. M120 group also enriched systemic lupus erythematosus and alcoholism (Figure 2B). In the M120 vs. M90 group, a total of 8 KEGG pathways (*P* < 0.05), including ECM-receptor interaction, focal adhesion, calcium signaling pathway, and cell adhesion molecules, were detected to be significantly rich in mRNAs with different expressions (Figure 2C). We detected four of these DEGs, MYH1 (gene ID: 102181426), NR4A1 (gene ID: 102185438), LOC102189516 (gene ID: 102189516), and TF (gene ID: 102172205), across all three comparisons. NR4A1 regulates DNA methylation and control of muscle differential development, which may be related to muscle proliferation and differentiation. In the first 50 DEGs of M120 vs. M90, we found that muscle differentiation markers MYH4 and CKM were highly expressed at day 120, and MYH2 and TNNT1 were associated with muscle contraction.

**Table 2:** Biological process enriched by the differentially expressed mRNAs in M120 vs. M90 group.

**GO terms**	***P*-value**	**Number of genes**
**Biological process**		
GO:0009725: response to hormone stimulus	1.58 × 10^−5^	16
GO:0033993: response to lipid	2.07 × 10^−5^	14
GO:0009719: response to endogenous stimulus	3.19 × 10^−5^	21
GO:0007155: cell adhesion	4.24 × 10^−5^	33
GO:0022610: biological adhesion	4.24 × 10^−5^	33
GO:0071396: cellular response to lipid	4.31 × 10^−5^	13
**Cellular component**		
GO:0042612: MHC class I protein complex	1.43 × 10^−5^	5
**Molecular function**		
GO:0005509: calcium ion binding	7.08 × 10^−10^	70

### Enrichment Analysis of the Differentially Expressed MiRNAs

**Figure 3:**
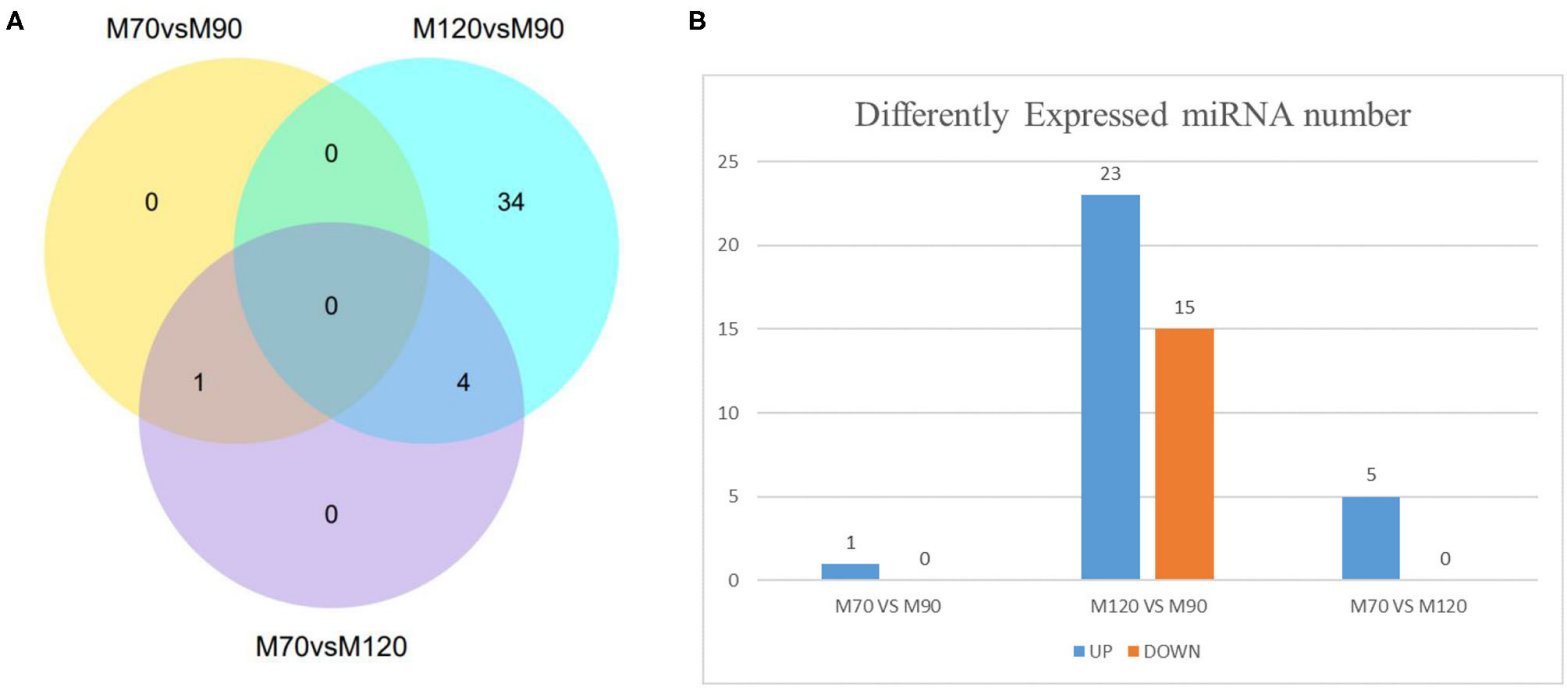
Number of DE-miRNAs at different time stage comparisons. **(A)** The venn diagram shows the number of DE-miRNAs across three comparisons: M70 vs. M90, M120 vs. M90, M70 vs. M120. **(B)** The number of up- and downregulated DE-miRNAs across three comparisons: M70 vs. M90, M120 vs. M90, M70 vs. M120, with the threshold set to *q* < 0.05.

**Figure 4:**
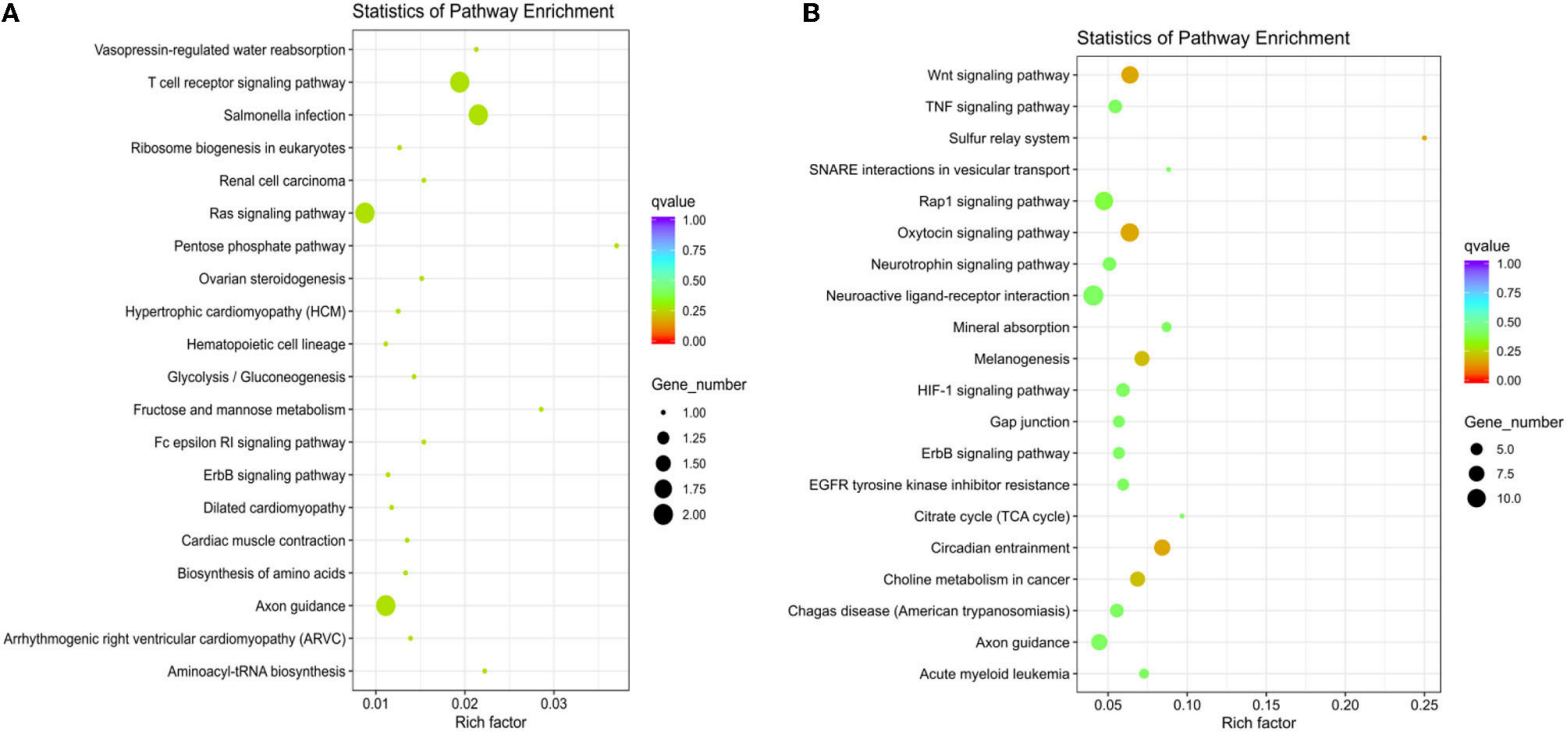
**(A)** The top KEGG enrichment analyses of the differentially expressed miRNAs in M70 vs. M120. **(B)** in M120 vs. M90.

According to the sequencing results, we found a total of 39 differentially expressed miRNAs, including 38 in the M120 vs. M90 group (Supplementary Material 4), 1 in the M70 vs. M90 group, and 5 in the M70 vs. M120 group (Figures 3A,B; Supplementary Materials 5, 6). The latter two groups did not significantly enrich GO terms, while in the KEGG pathway, the enrichment degree is not high and not significant (Figure 4A), and here we mainly analyzed group M120 vs. M90 (Supplementary Materials 7, 8). According to the results of GO terms, it mainly includes cell growth, regulation, response, protein dimerization activity, and sulfur compound binding (Table 3). Pathway analysis showed that differentially expressed miRNAs were enriched in 20 KEGG pathways, such as circadian entrainment, oxytocin signaling pathway, wnt signaling pathway, and sulfur relay system (Figure 4B).

**Table 3:** Biological process enriched by the differentially expressed mRNAs in M120 vs. M90 group.

**GO terms**	***P*-value**	**Number of genes**
**Biological process**		
GO:0016049: cell growth	0.00316	4
GO:0050678: regulation of epithelial cell proliferation	0.004178	3
GO:0032102: negative regulation of response to exteRNAl stimulus	0.005132	3
GO:0080135: regulation of cellular response to stress	0.006125	4
GO:0006952: defense response	0.006125	10
GO:0007187: G-protein coupled receptor signaling pathway, coupled to cyclic nucleotide second messenger	0.006151	3
GO:0007188: adenylate cyclase-modulating G-protein coupled receptor signaling pathway	0.006151	3
GO:0006954: inflammatory response	0.006834	6
GO:1901565: organonitrogen compound catabolic process	0.007362	6
GO:0050673: epithelial cell proliferation	0.00771	3
**Cellular component**		
GO:0005669: transcription factor TFIID complex	0.026849	2
GO:0044463: cell projection part	0.045274	3
GO:0031985: Golgi cisteRNA	0.045864	2
**Molecular function**		
GO:0019842: vitamin binding	0.002347	5
GO:1901681: sulfur compound binding	0.006201	4
GO:0046983: protein dimerization activity	0.006907	11

### Gene Network Analysis and Validation by Quantitative Real-Time Polymerase Chain Reaction (qRT-PCR)

**Figure 5:**
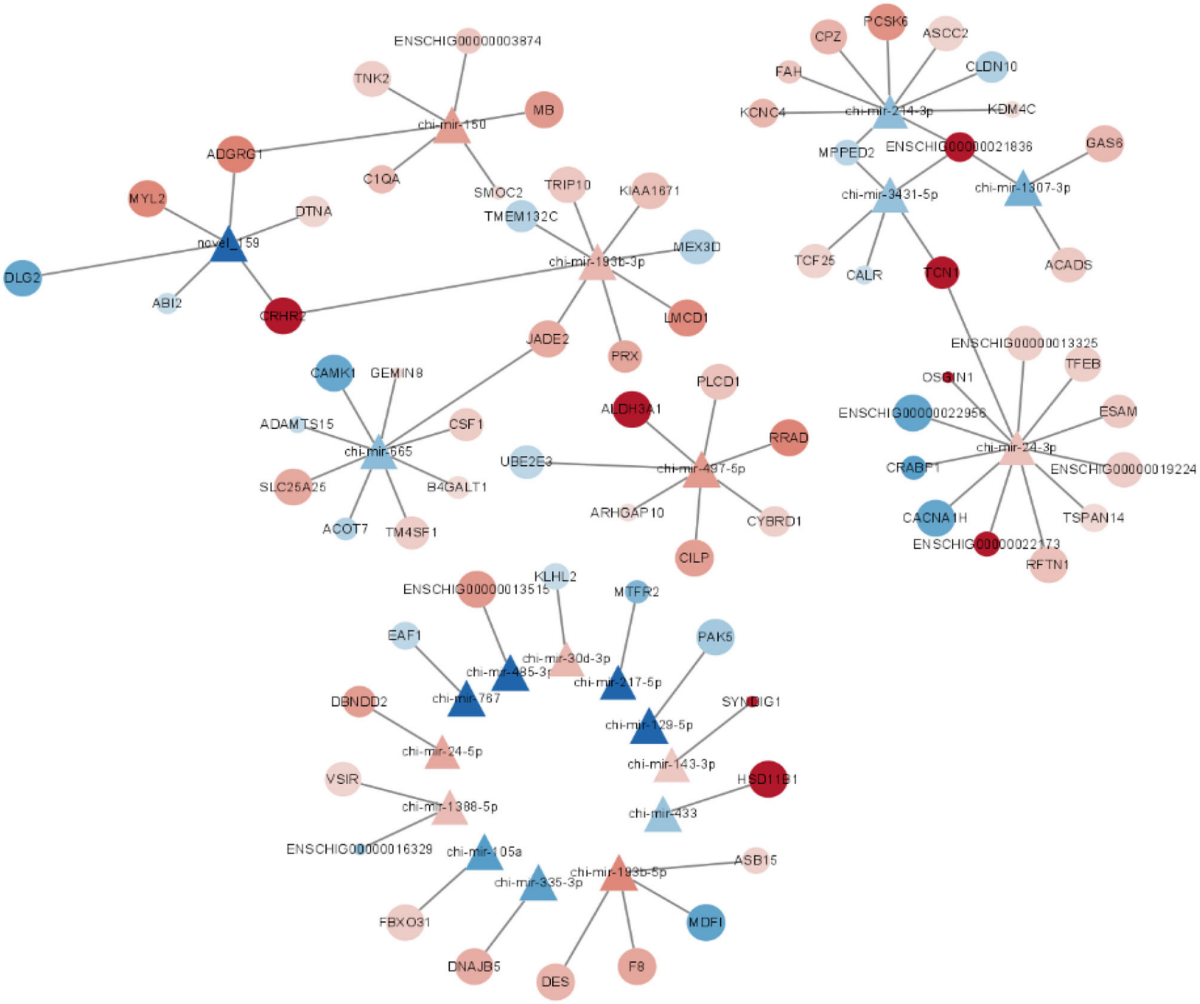
Interaction network map of the differentially expressed mRNAs targeted by M120 vs. M90 differentially expressed miRNAs.

**Figure 6:**
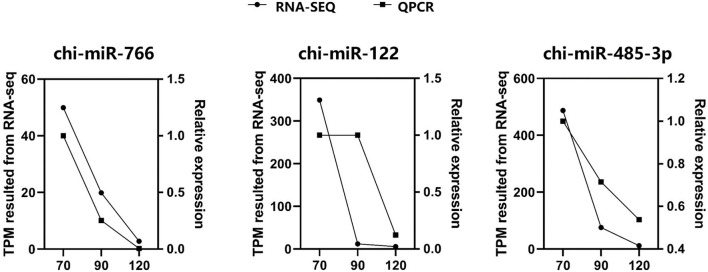
The expression level of three miRNAs were validated by qRT-PCR and compared with the results of RNA-SEQ. For qRT-PCR data, miRNA expression was normalized to U6.

Through miRNA target gene prediction by miRanda, DEmiRNA-mRNA interaction prediction was constructed, with a total of 81 DEmRNAs targetings 21 DEmiRNAs. Since DEmiRNAs in the 70–90 and the 70–120 days were the same as those in the 120–90 day group, only 120–90 day data were used to construct the targeted network. We can find these differentially expressed miRNAs whose target genes do not overlap much (Figure 5). Among them, chi-miR-24-3p, chi-miR-497-4p, chi-miR-665, chi-miR-193B-3p, and chi-miR-214-3p are DEmiRNAs mainly targeted by DEmRNAs. Most DEmiRNAs and DEmRNAs were up-regulated with gestation date. A total of 3 DEmiRNAs were selected for verification by qRT-PCR. Based on the qRT-PCR results, the expression of these 3 DEmiRNAs was consistent with that in the RNA-SEQ results (Figure 6). The expression of chi-miR-122 is different between RNA-SEQ and qPCR results in 90 days, which may be caused by the difference in miRNA expression between individual goats, but the overall expression is still down-regulated.

## Discussion

With the advancement of high-throughput sequencing technologies, non-coding RNAs are becoming increasingly important in biological development. MiRNA, a kind of non-coding RNA, has been found to have a role in skeletal muscle development and differentiation. With the rise of the animal husbandry business in recent years, several research teams have begun to look into the control of sheep and goat muscle growth and the production of non-coding RNA. Ling, Y identified miRNAs in skeletal muscle of five fetal and two childhood goats by RNA-SEQ, identified 421 known miRNAs and 228 new miRNAs in total, and explored the temporal expression profile of skeletal muscle development in goats (39). Liao, R constructed mRNA and miRNA expression profiles of Chongming white goats and found that chi-miR-206 and chi-miR-133a/B were associated with muscle development, and Mir-99b-3p, 224, and 10B-5p were highly expressed in 7-month-old muscle tissue (40). Li et al. found that goat Mir-101A, as a new myogenic micro modification of myogenic differentiation, was up-regulated in the process of satellite cell differentiation (17). Ling et al. found that Mir-27B in goat muscle regulates myogenic proliferation and differentiation by targeting PAX3 (41). Yuan et al. sequenced sheep longissimus dorsi muscle at multiple development time points and detected that miR-2387, miR-105, miR-767, miR-432, and miR-433 were widely expressed during embryonic development (42). These results suggest that different miRNAs play different embryonic skeletal muscle development stages.

The prenatal stage is crucial for skeletal muscle development in ruminants since nearly all muscle fibers are produced during this time rather than after birth (43). We chose F70, F90, and F120 for our investigation since previous research has indicated that miRNA expression in goats is particularly active throughout pregnancy and maybe in the active period of differentiation around 70–120 days. In all three time periods, the expression levels of muscular-specific miRNA miR-1, 133, and 206 were quite high. Mir-1's key target genes are HDAC4, PAX3, PAX7, NotCH3, HDAC2, ND1, and COX1 (44), according to previous research. Mir-133 overexpression enhances myoblast proliferation while preventing differentiation. MiR-133 and mir-206 were expected to block DNA polymerase and enhance muscle development with FSTL1 and UTM, according to knockout tests (16). Therefore, we can speculate that muscle proliferation and muscle differentiation exist simultaneously in Leizhou goats from day 70 to day 120. We found that the expression of miR-129-5p decreased sharply from day 70 to day 120 in DEmiRNAs (*p* < 0.05). In the latest studies, miR-129-5p can negatively regulate myogenic differentiation and inhibit the expression of myogenic genes such as MyOD, MyOG, and MyHC. MiR-129-5p targets the 3 ‘UTR region of MEF2A and is repressed in C2C12 cells (45). Mir-129-5p mainly enriches in the cytoskeleton, hydrolase activity, protein binding, and tetrapeptide duplication in GO. We predicted that the target gene of miR-129-5p, GPRC5B, was identified as a retinoic acid-induced protein that plays an important role in the proliferation and differentiation of multiple cell lines. GPRC5B enhanced collagen production in myofibroblasts and promoted tissue fibrosis (46). MiR-433 was previously shown to rescue impaired myogenic differentiation of C2C12 cells, increasing myoduct formation and myosin heavy chain (MHC) expression (47). In our study, HSD11B1, the target gene of miR-433, was highly expressed at 90–120 days, and the expression and activity of HSD11B1 increased with myoduct differentiation and glucocorticoid action in mouse skeletal muscle studies (48). Hsd11b1 is involved in oxidoreductase activity, single-organism metabolic process, catalytic activity, etc. We speculated that muscle tube differentiation was also occurring in Leizhou goats during this period. MiR-24-3p can control the myogenic differentiation and proliferation of skeletal myogenic cells in fetal cattle (49). In our results, CACNA1H, the target gene of miR-24-3p, was decreased during 90–120 days. CACNA1H enriches a variety of channel activities and transmembrane transport. Previous studies have found it to serve as a key regulator in maintaining a muscle's complex regulatory network (50).

On the other hand, we found that some muscle differentiation markers were differentially expressed at 120–90 days. Creatine kinase, M-Type (CKM), CK catalyzes the reversible ATP-dependent interconversion of creatine into phosphocreatine (PCR), building up a pool of rapidly diffusable PCR for spatiotemporal buffering of ATP levels (51). Thus, CK plays a pivotal role in tissues with high and fluctuating energy demands such as muscle. In many muscle studies, CKM has been used as a marker of myogenic differentiation and muscle integrity (52). In our study, CKM expression decreased rapidly at 70–90 days and was heavily expressed at 120 days, so we speculated that there was skeletal muscle differentiation in 90–120 days or even after 120 days. MYH3, one of the isoforms of Myosin Heavy Chain (MyHC), is also a marker of myogenic differentiation (53). Stern-straeter confirmed the correlation between the differentiation marker of the MYH3 gene and creatine kinase (CK) activity (52), and F NIU found that MYH3 had a potential influence on growth performance and carcass traits of Qinchuan cattle (54). Sequencing showed that the expression of MYH3 was gradually down-regulated but still at a high level during this period. Furthermore, we discovered that ITGB4, VWF, COL4A4, ITGA11, LAMA3, RELN, COL6A3, LAMB2, COL4A1, ITGA4, COL27A1, TNC, COL24A1, ENSCHIG00000014869, ENSCHIG00000022140, and ENSCHIG00000017158 were all enriched in ECM (Extracellular Matrix)-receptor interaction, Focal adhesion, and the PI3K-Akt signaling pathway in M120 vs. M90 group. ECM is required for various cellular responses, including transcription, inflammation, proliferation, and differentiation (55). Focal adhesion regulates skeletal muscle development and is involved in several critical signal pathways (56), including the WNT signaling route, the MAPK signaling pathway, and the PI3K-Akt signaling pathway. It is worth emphasizing that the connection between Focal adhesion and ECM regulates several intracellular pathways of cell motility, proliferation, and differentiation (57). The PI3K-Akt signaling pathway has also been shown to increase skeletal muscle cell proliferation and differentiation (58, 59). We hypothesize that these differentially expressed genes enriched in these three signal pathways are engaged in skeletal muscle differentiation functions, resulting in differential expression.

## Conclusion

In conclusion, goat muscle differentiation is a complex process coordinated by many miRNAs and mRNAs. Combined with this study, high expression of muscle-specific miRNA, miR-1, 133, 206, differential expression of miR-129-5p, miR-433, miR-24-3p, and muscle-specific mRNA CKM and MYH3, we can confirm that muscle differentiation exists in Leizhou goats at 90–120 days. Understanding the miRNA and mRNA that may affect muscle differentiation of Leizhou goats screened by us will be helpful to animal breeding of Leizhou goats and have a particular significance for improving the meat yield of Leizhou goats.

## Data Availability Statement

The datasets presented in this study can be found in online repositories. The names of the repository/repositories and accession number(s) can be found in the article/Supplementary Material.

## Ethics Statement

The animal study was reviewed and approved by Ethics Committees of the Laboratory Animal Center of South China Agricultural University (Permit number: SYXK-2014-0136).

## Author Contributions

XZo and YL: sample collection. JY: data curation. YG and YL: methodology. GL and DL: project administration. GL and BS: software. YG, MD, and YL: supervision. HX and JY: validation. JY and XZh: writing—original draft. YL: writing—review and editing. All authors have read and approved the manuscript.

## Funding

This research was supported by the Modern Agricultural Industrial Technology System of Guangdong Province (2021KJ127).

## Conflict of Interest

The authors declare that the research was conducted in the absence of any commercial or financial relationships that could be construed as a potential conflict of interest.

## Publisher's Note

All claims expressed in this article are solely those of the authors and do not necessarily represent those of their affiliated organizations, or those of the publisher, the editors and the reviewers. Any product that may be evaluated in this article, or claim that may be made by its manufacturer, is not guaranteed or endorsed by the publisher.
